# Expanding mental health support: Empowering medical professionals with greater resilience

**DOI:** 10.4102/sajpsychiatry.v31i0.2386

**Published:** 2025-01-15

**Authors:** Yilin Jiang, Heng Zhou, Narina A. Samah

**Affiliations:** 1 School of Education, Universiti Teknologi Malaysia, Skudai, Malaysia; 2 Department of Student Affairs, Chongqing Medical University, Yuzhong, China

Dear Editors

We conducted a review of the study titled ‘The Effects of Inpatient Suicide on Nurses at Weskoppies Hospital: A Qualitative Study’.^1^ This research reveals the psychological and emotional toll that inpatient suicides impose on nursing staff. The study’s findings reveal the profound impact these traumatic events have on nurses, who are often the first responders. As highlighted, nurses experience a wide array of emotions, including guilt, fear, and sadness, which can persist long after the event, affecting their mental health, professional performance, and overall well-being. The qualitative approach adopted in this study provides an in-depth exploration of nurses’ personal experiences that quantitative data alone cannot capture.

While this study holds substantial significance, there are aspects that could benefit from additional refinement in future research. The selected qualitative research paradigm emphasises reciprocity, suggesting that researchers and participants should experience growth during and after the study.^2,3^ Although counselling was arranged for participants post-study, the study could have benefited from reporting on growth experiences during the process. Furthermore, qualitative research greatly emphasises the researcher’s reflexivity and cultural sensitivity.^3^ If the researcher shares more personal experiences and cultural background, it will enable readers to understand the foundations of the conclusions better. In addition, qualitative research is often critiqued for its perceived lack of transparency.^4,5^ Therefore, demonstrating the coding process would give readers a clearer understanding of the study’s content and methodology.

Psychiatric nursing units, among other departments, are severely impacted by the high rates of suicide among psychiatric patients.^1,6^ Research suggests that healthcare professionals who are exposed to patient suicides exhibit an increased vulnerability to mental health challenges.^7^ Therefore, it is essential to implement a comprehensive approach to support them, such as establishing clear, step-by-step emergency protocols that provide immediate psychological support to both patients and staff involved in or witnessing a suicide attempt.^8^ Moreover, regular and timely communication with peer hospitals and relevant medical education institutions should be established to improve the management of these events by enabling data sharing on risk factors, standardising assessment protocols, and developing early warning systems. Furthermore, promoting the rapid adoption of best practices, particularly through integration into early medical education, will enhance the identification of patients at risk of suicide, expedite response times, and strengthen the resilience of medical professionals in handling such cases.

In conclusion, this article serves as a vital reminder of the frequently neglected emotional burden carried by nursing staff in psychiatric settings. By making mental health and well-being of medical professionals a priority, healthcare institutions can empower their staff with greater resilience and enhance their ability to deliver high-quality care. This approach is crucial for patient recovery and overall healthcare outcomes.
